# *In vitro* larvicidal effects of ethanolic extract of *Curcuma longa* Linn. on *Haemonchus* larval stage

**DOI:** 10.14202/vetworld.2016.417-420

**Published:** 2016-04-28

**Authors:** Norisal Binti Nasai, Yusuf Abba, Faez Firdaus Jesse Abdullah, Murugaiyah Marimuthu, Abdulnasir Tijjani, Muhammad Abubakar Sadiq, Konto Mohammed, Eric Lim Teik Chung, Mohammed Ariff Bin Omar

**Affiliations:** 1 Department of Veterinary Clinical Studies, Faculty of Veterinary Medicine, Universiti Putra Malaysia, 43400 UPM Serdang, Selangor, Malaysia; 2 Department of Veterinary Pathology and Microbiology, Faculty of Veterinary Medicine, Universiti Putra Malaysia, 43400 UPM Serdang, Selangor, Malaysia; 3 Research Centre for Ruminant Disease, Faculty of Veterinary Medicine, Universiti Putra Malaysia, 43400 UPM Serdang, Selangor, Malaysia

**Keywords:** *Curcuma longa*, ethanolic extract, *Haemonchus*, larvae 3, levamisole, strongyle

## Abstract

**Aim:**

Gastrointestinal helminthosis is a global problem in small ruminant production. Most parasites have developed resistance to commonly available anthelminthic compounds, and there is currently an increasing need for new compounds with more efficacies. This study evaluated the *in vitro* effects of ethanolic extract of Curcuma longa (EECL) as a biological nematicide against third stage Haemonchus larvae (L3) isolated from sheep.

**Materials and Methods:**

Haemonchus L3 were cultured and harvested from the feces of naturally infected sheep. EECL was prepared and three concentrations; 50, 100, and 200 mg/mL were tested for their efficacies on Haemonchus L3. Levamisole at concentration 1.5 and 3 mg/mL were used as positive controls.

**Results:**

EECL showed anthelmintic activity in a dose-dependent manner with 78% worm mortality within 24 h of exposure at the highest dose rate of 200 mg/mL. There was a 100% worm mortality rate after 2 h of levamisole (3 mg/mL) admisntration. However, there was a comparable larvicidal effect between when levamisole (1.5 mg/mL) and EECL (200 mg) were administered.

**Conclusion:**

The study shows that EECL does exhibit good anthelmintic properties at 200 mg/mL which is comparable with levamisole at 1.5 mg/mL.

## Introduction

Gastrointestinal helminths are among the most significant factors causing retarded growth in ruminants. Helminthosis is also one of the growing threats to the livestock production worldwide [1]. Treatment of helminthosis has become worrisome over the years due to the development of resistance by the parasites to chemical drugs available commercially in the market. Thus, there is an increasing need for alternative natural compounds that are safe and effective in combating this menace. Turmeric has been investigated widely and is said to exhibit different properties such as anti-inflammatory, hypercholesterolemic, choleretic, antimicrobial, insect repellent, antirheumtaic, antifibrotic, antivenomous, antiviral, antidiabetic, and antihepatotoxic as well as anticancerous properties [2,3].

The antiparasitic effect of curcumin, which is one of the active compounds in *Curcuma longa* has been observed to be dose-dependent, with higher concentrations of the compounds exhibiting the greatest effects. However, the exact mechanism of its action is still poorly understood and need to be studied in more detail [4]. Curcumin extract has been found to be effective against *Shistosoma mansoni* and earthworm muscle cells in a dose-dependent manner [5,6]. Its efficacy in *Ascaridia galli* in chicken has also been evaluated both *in vitro* and *in vivo* with success [7].

This study was designed to evaluate the effect of ethanolic extract of *C. longa* (EECL) as a treatment alternative for gastrointestinal heminths (strongyles) of sheep. The study will also evaluate the comparable efficacies of *C. longa* and levamisole on the survival rate of *Haemonchus* third stage larvae (L3) *in vitro*.

## Materials and Methods

### Ethical approval

Ethical approval was not required for this study as only fecal samples were collected from naturally infected sheep.

### Preparation of plant materials

*C. longa* (turmeric) tubers were collected from the field in Serdang area. These tubers were thoroughly washed in water, cut into smaller pieces and left to dry at room temperature for 4 days. The dried turmeric was ground to powder form with a mortar and pestle and stored at room temperature in sealed plastic bags until it was used.

### Preparation of EECL (turmeric)

Ethanolic extract of curcuma was prepared as previously described by Salama *et al*. [8]. Briefly, the fresh curcuma rhizomes were washed, dried, ground into powder, and weighed. 100 g of the powder was dissolved in 1 L of 95% ethanol at a ratio of 1:10 and left for 3 days at 25°C. The mixture was shaken at 4-6 h intervals. Filtration was done, and the liquid was evaporated to form a concentrated extract. This was kept in an incubator for a further 72 h to evaporate the residual. The sediment was then diluted with distilled water at a ratio of 200:1 to prepare the extract stock solution (200 mg/mL). Concentrations ranging from 100, 50, and 25 mg/mL were prepared by dilution of the stock solution with distilled water.

### Preparation of levamisole concentrations

The initial concentration of levamisole was 32 mg/mL (Nilverm^®^ Oral Drench, India) and was diluted with distilled water to get concentrations of 1.5 and 3.0 mg/mL.

### Fecal culture and harvesting of third larval stage (L3) Haemonchus

Fecal samples were collected from naturally infected sheep (containing <250 epg), crushed and placed in a clean glass jar. The moisture of the feces in glass jar was maintained everyday by putting a few drops of distilled water for 7 days. A few scoops of charcoal were mixed with fecal material collected from diarrhoeric animals to prevent excessive moisture. After 7 days, the L3 stages were harvested from the culture by filling the glass jar with warm distilled and inverting the glass jar on a petri dish for 30 min. The L3 was observed under a dissecting microscope and harvested into a clean glass bottle and stored in the refrigerator at 4°C. Identification of the worm larvae was done by putting them on a glass slide with one drop of Lugol’s iodine added before putting a cover slip onto it and examining under light microscopy.

### Treatment and motility assessment

About 50 active L3 were put in a petri dish and maintained with 8.0 mL distilled water. The L3 were then treated with 1.0 mL *C. longa* extract at 200, 100, and 50 mg/mL. 1.0 mL levamisole at concentration 1.5 and 3 mg/mL was used as positive control and 1.0 mL distilled water was used as negative control. The petri dishes were shaken manually for 1 min and were kept at room temperature. Each Petri dish was examined every 2, 4, 6, and 24 h post-treatment. 4 replicates were done for each treatment. For every 2 h, the motility of the L3 was checked and recorded for each motile and non-motile or dead L3. The dead L3 were confirmed by observing the absence of motility for up to 10 s. The mortality index formula was used to determine the rate of L3 mortality.


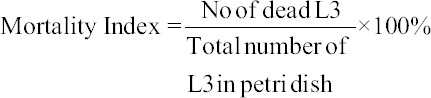


### Statistical analysis

Statistical analysis was performed using SPSS version 20.0 statistical software. Results were assessed for its normality. One-way ANOVA was used for normally distributed data and p<0.05 was considered to be significant. Independent sample t-test was used for data in two independent or unrelated groups that have different means.

## Results

### Parasite larvae identification

Before the experiment was conducted, identifications were done on 100 strongyles that were harvested from the fecal culture by microscopic examination as previously described [9]. Based on the examination, the harvested strongyles comprised 97% *Haemonchus contortus* and 3% *Oesophagostomum* sp.

### Effects of turmeric on Haemonchus L3 Viability

**Figure 1:**
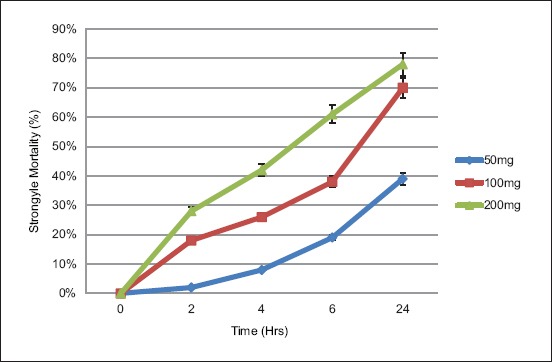
Percentage of larvae mortality at different hours following treatment with different concentrations of ethanolic extract of tumeric.

**Figure 2:**
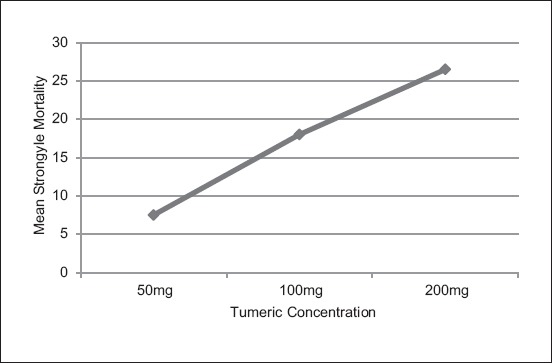
Mean non-motile strrongyle L3 following treatment with different concentrations of ethanolic extract of tumeric.

The different effects exhibited by different concentration of turmeric on the *Haemonchus* larvae mortality are shown in Figure-1. There was an increase in the number of dead larvae over time as turmeric concentrations exhibited the highest antihelmintic activity in a dose-dependent way with the maximum effect observed at the highest dose of turmeric extract; 200 mg/mL, where 78% of the worms died 24 h post-exposure. The mean mortality of larvae showed that it increased as turmeric concentration increased (Figure-2). The LC_50_ for MECL was estimated to be 128.2 mg, while the LC_90_ was 231 mg. The mortality of larvae was compared to the levamisole as the reference drugs or positive control at concentration of 1.5 mg/mL at 24 h and 3.0 mg/mL at 2 h post-exposure. At 1.5 mg/mL of levamisole, 72% larvae mortality was observed at 24 h post-exposure, while at 3.0 mg/mL, 100% larvae died at 2 h post-exposure. There was no mortality of larvae observed in distilled water (negative control) at 24 h post-exposure.

### Comparative effects of turmeric at 200 mg/mL against levamisole at 1.5 mg/mL

**Figure 3:**
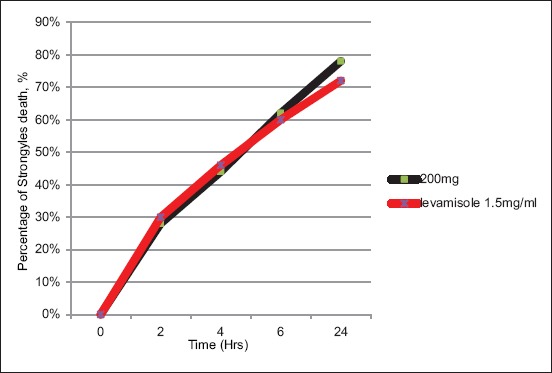
Comparison of larvae mortality at different hours following treatment with 200 mg/mL of ethanolic extract of tumeric and 1.5 mg/mL of levamisole.

The effect of turmeric extract was compared at 200 mg/mL with levamisole at 1.5 mg/mL. The result showed that there is no significance difference (p<0.05) between the two concentrations over a course of 24 h post-exposure (Figure-3).

## Discussion

Tumeric has been long shown to exhibit different properties [2]. Turmeric hydroalcoholic extracts have shown a remarkable anthelmintic potential against intestinal parasitism [10]. According to Kulkarni *et al*. [11], a maximum concentration of curcuminoids was obtained in methanol extract in the form of a dark black orange color compared to other extraction solvent such as acetone, chloroform, and ethyl acetate. This shows that methanol is a good solvent to be used to extract a high concentration of curcumin content compared to any other solvent.

In this study, we observed that the efficacy of ethanolic extract of tumeric increased in a time and dose-dependent manner, as the number of larvae mortality increased with increasing dose and time. This could be due to the presence of more cucurminoid content in the extract as the concentration increased from 50 to 200 mg. Effects of *C. longa* as an anthelmintic revealed that some parts of cucurbits possess anthelmintic properties due to secondary metabolites such as cucurbitacin [1]. Previous studies reported that worms exposed to turmeric extract were paralyzed and died in a time and dose-dependent manner. The effect of turmeric extract on earthworm muscle cells was also found to be dosage dependent [5]. Similarly, Magalhães *et al*. [6] observed that curcumin at 50 and 100 µM caused 100% mortality in adult *S. mansoni* and at 5 and 20 µM decreased worm viability in comparison to negative control. The authors also reported that 5 and 10 µM reduced egg production by 50%, which was recently associated with transcriptional repression observed in Notch and transforming growth factor-β pathways [12].

With an increasing problem of anthelmintic resistance in small ruminants, especially sheep, there is an increasing demand to find alternative treatment for the helminthosis. According to Mohammed *et al*. [13], *H*. *contortus* is the major cause of small ruminant internal parasitism in warm and moist climatic regions. This is true as we observed 97 out of 100 isolated strongyles larvae to be *H. contortus* and only 3 were *Trichostrongylus* sp.

There was no significance difference between the effects of levamisole at 1.5 mg/mL and turmeric extract at 200 mg/mL on the mortality rate of larvae after 24 h of treatment. Levamisole kills worms by depolarizing nicotinic acetylcholine receptors in muscular junction and cause paralysis and death of worms [14,15]. While for turmeric, the curcuminoid is believed to be the main component that causes mortality of the worms, perhaps in a similar manner to that of levamisole. As stated earlier, a study by Vidya *et al*. [5] showed turmeric extract to have good growth suppression on earthworm muscle cells. Since nematodes or helminthes are only one level lower than earthworms, it may be considered that turmeric extract has similar effects on nematodes as it did on earthworms. Curcumin is a natural polyphenol that is responsible for antiproliferative activity on dividing cells; this activity may have caused the muscle growth suppression in earthworm. Although the antiparasitic effect of curcumin is more obvious at a higher concentration, the exact mechanism of action is not yet fully understood and may vary depending on the helminth parasite [4].

## Conclusion

Turmeric extract at 200 mg/ml showed the highest anthelmintic properties with 78% mortality in L3 within 24 h. Similarly, it also showed a comparable effect at this dose with 1.5 mg/mL of levamisole. It can thus be concluded that the EECL exhibits good anthelmintic activity against *Haemonchus* larvae and can serve as a potential substitute for levamisole.

## Authors’ Contributions

MM, FFJA, and MAO designed and conceptualized the work. YA, AT, MAS, NBM, KM, and ELTC conducted the work and drafted the manuscript. All authors have read and approved the final manuscript.
